# A New Patent Electrolyser

**Published:** 1917-05-19

**Authors:** 


### A NEW PATENT ELECTROLYSER.

For many years the sterilisation of water by means of electric current and by bleaching powders (chloride of lime) has received close attention from chemists and scientists. Messrs. Vincent Roberts and Co., of Leeds, have recently introduced a new patent electrolyser, which they claim is suitable for use on board ship and in hospitals. The apparatus consists of a rectangular box of porcelain, divided by electrodes into a number of compartments, so arranged that "brine entering at the highest point flows by gravity under and over the electrodes, until it passes out at the lowest end changed into hypochlorite, running finally into a carboy, where it is stored for use."

### Working.

"The passage of a current of electricity through water decomposes it, setting free hydrogen and oxygen. If now common salt be added to the water, the process becomes complicated, as, in addition to the electrolysation of hydrogen and oxygen, chlorine and sodium are set free, and they instantaneously make fresh combinations, one being the *combination of chlorine with the oxygen to form hypochlorous acid.*"

The patentees claim that the apparatus has been largely used in laundries, with gratifying results, inasmuch as linen lasts longer, is of better colour, and is sterile; while on the launderers' part the addition of the electrolytic sodium hypochloride to soap and water in the washing-machine promotes lathering and saves soap. The apparatus is stated to be also of commercial value for bleaching purposes in mills, and it is claimed that vegetable fibres bleached with it are of 10 per cent. greater tensile strength than when bleached with chloride of lime. From a hospital point of view, it is known that hypochlorous acid is a potent germicide, utterly destroys bacteria, dispenses with other and more expensive antiseptics, and its use promotes the rapid healing of wounds. For ships' use, the electrolysation of sea-water by the apparatus renders the water efficient for hygienic sterilising and for laundry work.

As we have pointed out, the use of electrolysers is not new, but our inquiries have shown that their use in land laundries is not at all general; in hospital laundries the apparatus does not seem to have been adopted to any extent, if at all. This negative statement does not imply that their use might not be advantageous, but that the idea seems not yet to have passed from an experimental stage of scientific procedure to general use. It should be borne in mind that the introduction of an apparatus of the kind means additional work for the laundry and additional responsibilities for the laundryman. The present time, therefore, when all institutions are working at the fullest pressure and their laundries are short staffed, does not seem a favourable one to introduce additional machinery, although such apparatus may, in the long run, prove serviceable. Also, it must not be overlooked that the solution of electrolytic sodium hypochlorite rapidly loses its strength by decomposition, is affected by light and air, and will not allow of much movement. Small quantities alone, made up as required, could therefore only be advantageously employed, and this would mean more or less continuous use of the apparatus. Above all, it is essential that the claim that linen lasts longer, and the acid has no deleterious action, should be supported by confirmatory evidence. Also, as flannels are a very considerable part of an institution's washing output, it will be necessary that similar evidence be adduced that such articles will not be affected by bleaching or otherwise.

### The Electrolyser as a Bactericide.

As a bactericide the researches of Professor Lorrain Smith, Professor J. M. Beattie, Professor Dakin, and others have done much to bring the use of hypochlorous acid into prominence. We believe that these gentlemen claim, after much scientific experimentation, that the acid may ultimately become generally used in hospitals and institutions, to the discontinuance of more expensive and, in their opinion, less efficient antiseptics now generally used. If experience supports their investigations, there can be no doubt that a great saving will for a time be affected in hospitals and kindred institutions. We say for a time, because the persistent research which is being carried on will necessarily mean the displacement from time to time of props on which reliance has been placed. If, as we believe, the new electrolyser will provide institutions with a daily supply of hypochlorous acid for use as an antiseptic, the apparatus is undoubtedly worthy of careful attention.

### Hygiene.

It is claimed for the apparatus that it can be used on board ship for hygienic purposes, and in view of experiments lately carried out by the Medical Research Committee appointed by the Government to inquire into the value of electrolysed sea-water, the apparatus is doubly deserving of careful attention. Careful tests of electrolysed sea-water were recently made on hospital ships coming from the Dardanelles with cases of enteric and dysentery, the instructions being for regular flushing of the ships with the solution, and for close observations and bacteriological tests to be made. We understand these tests were satisfactory, and showed the solution to be of great value as a bactericide and disinfectant. Full particulars of these and other tests will, however, doubtless be published in due course by the Medical Research Committee in their reports, and if these are satisfactory the claims of the makers of the present apparatus will be considerably enhanced.

Generally, our inquiries among bleachers, dyers, spinners, and other manufacturers go to show that, while an open mind exists as to the introduction of electrolysers, a good deal of experience must yet be forthcoming before their use becomes general.

Time and accumulation of knowledge go hand in hand. Everyone should be the gainer, therefore, for taking into consideration the claims of the new patent electrolyser.

